# Emerging scientists in analytical sciences: Niklas Geue

**DOI:** 10.1002/ansa.202300049

**Published:** 2023-10-09

**Authors:** Niklas Geue

**Affiliations:** ^1^ Michael Barber Centre for Collaborative Mass Spectrometry, Manchester Institute of Biotechnology, Department of Chemistry The University of Manchester Manchester UK

Through a collection of editorials titled “Emerging Scientists in Analytical Sciences,” we aim to spotlight promising individuals who are actively engaged in the realm of analytical sciences. For this editorial, we invited Niklas Geue who recently submitted his PhD thesis at The University of Manchester (UK). We are keen for anyone working in this field to nominate somebody for a Q&A by sending an email to one of the editors and explaining to us why this person should be highlighted.

## 1 HOW DID YOU GET INVOLVED IN THE FIELD OF ANALYTICAL SCIENCES?

I grew up in Magdeburg, a middle‐sized city in East Germany, and went to a high school with a focus on maths, science, and technology. Thereby, I was exposed to a lot of science, and early on I participated in competitions, seminars, and other science events. My main interest was always chemistry, evidenced by a considerable lab in my grandparent's garage — much to everyone's annoyance. In my late high school years, I also participated in the International Chemistry Olympiad and made it to the final German selection round twice (among the best 16). The question of what I wanted to study was never really in doubt.

**FIGURE 1:**
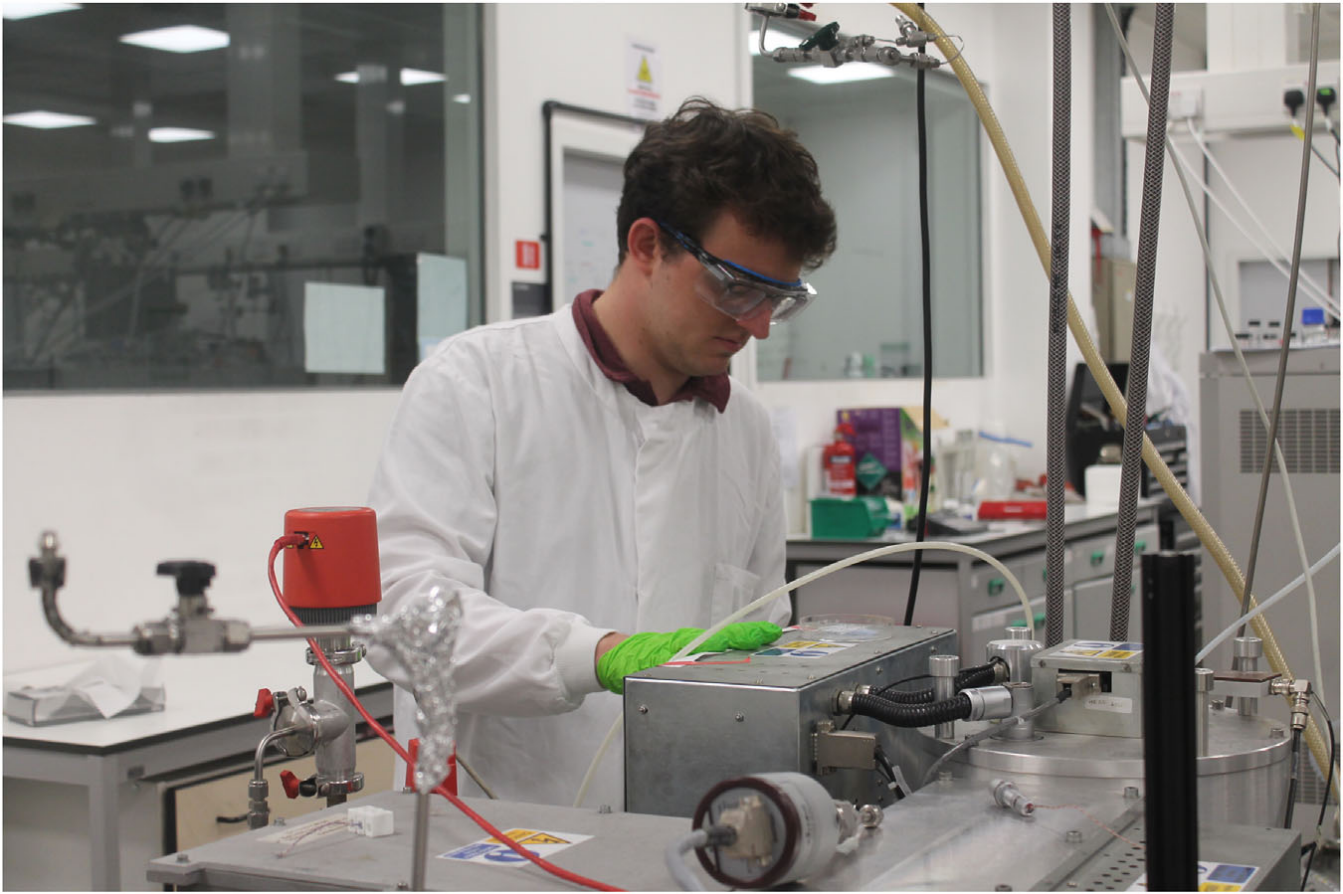
Niklas in the laboratory of the Michael Barber Centre for Collaborative Mass Spectrometry at The University of Manchester, September 2023. Photo: Aryaditi Jena.

For my Bachelor's I went to Leipzig, a great student city, and graduated as the best student of my year. During and following my undergraduate years, I undertook three research internships. These experiences took me to diverse locations around the world: one internship was based in Santiago de Chile focusing on kinetics/spectroscopy (related to my Bachelor's thesis), another in Sydney centred around mass spectrometry (MS), and a third in Los Angeles, where I further worked on my spectroscopic skills. During these research stays, I realized two things: my strong inclination to remain within the realm of analytical and physical chemistry and my eagerness to actively engage in research at the earliest opportunity. The UK was ideally suited for the latter as I could start my PhD here directly after my Bachelor's. I was also always fascinated by how things work on a molecular level, and similarly enthusiastic about the interdisciplinarity with instrumentation and engineering. I became very interested in MS while I was in Australia, and decided that I wanted to stay in this field for my PhD work (Figure 1).

## 2 WHAT IS THE TOPIC OF YOUR PhD STUDY?

My PhD project is about the characterisation of metallosupramolecular complexes using advanced MS techniques. These and similar molecular architectures are important in a range of fields (e.g., catalysis, medicine, and materials), and quite prominent, not just since the Nobel prize for molecular machines in 2016. Unfortunately, it is not straightforward to structurally characterise them properly.^1^ MS, particularly in combination with tandem MS and ion mobility (IM), is a great tool to enhance our understanding of such assemblies, by probing their stability as well as their size and shape.

**FIGURE 2:**
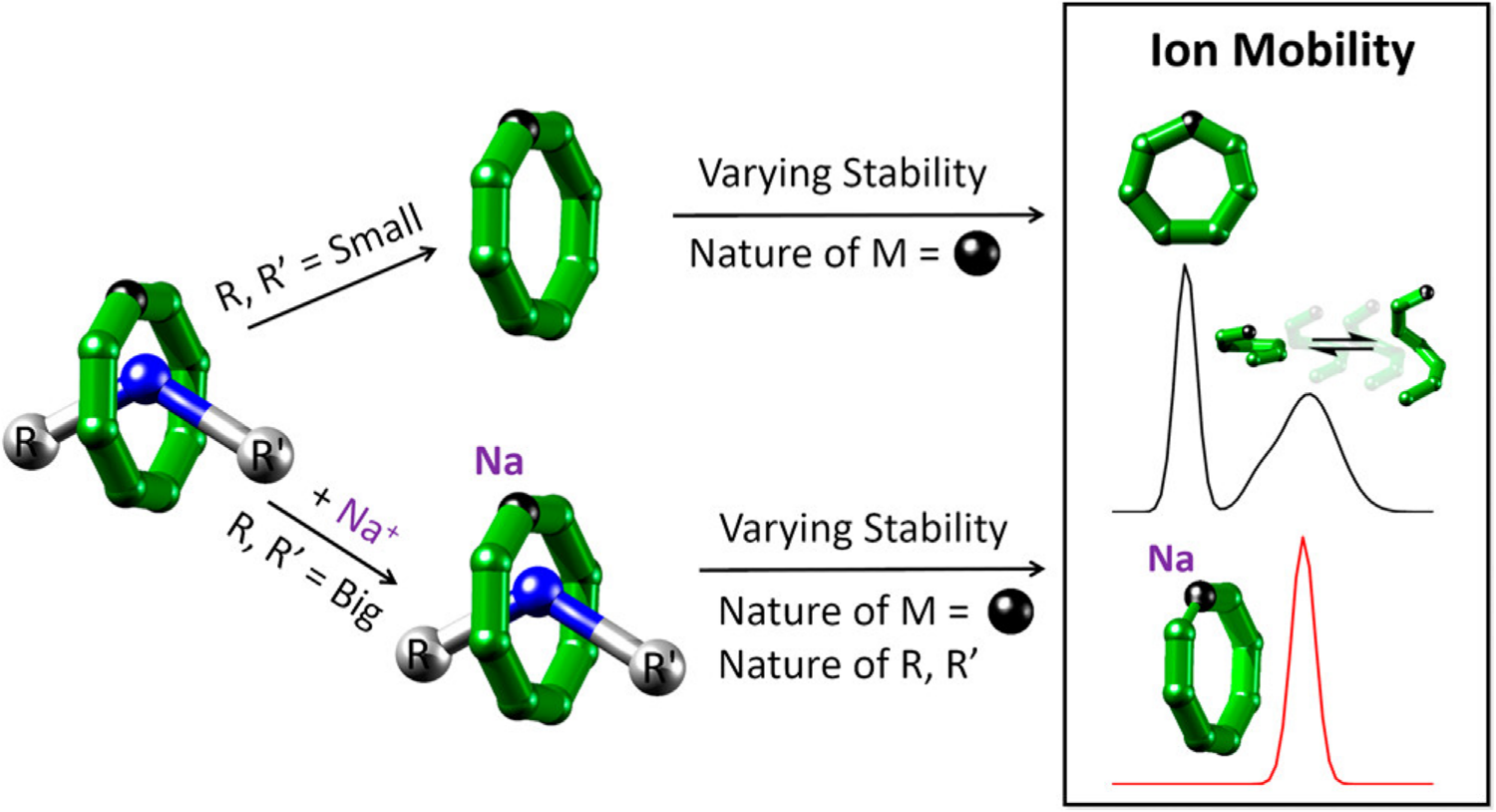
Graphical abstract illustrating how ion mobility mass spectrometry can track the disassembly mechanisms of polymetallic rings and rotaxanes as well as reveal the factors contributing to their stability. Understanding their stability and disassembly is crucial feedback for the synthesis of these molecules, which are relevant in electronics, materials, and quantum applications. Reprinted^2^ with permission.

During my PhD, I have successfully shown that it is possible to evaluate the stability of (metallo)supramolecular compounds using tandem MS, and I have used this methodology to systematically examine how the substitution of d‐metals, ligands, and charge carriers alter this property.^2^, ^3^ I was able to discriminate competing disassembly mechanisms, uncovering trends for closed versus open systems and small versus large polymetallic ions (Figure 2).^2^, ^4^ These results provided new insight into the contested criteria a threaded supramolecular assembly must fulfill to be considered a rotaxane, which is when the thread in the centre of the ring cannot slip off (Figure 2).^2^ I have also correlated computationally derived structures, using density functional theory (DFT), with experimental IM‐MS data to propose atomically resolved structures,^2^ and demonstrated how different charge‐carrying ions can measure the cavity size of polymetallic complexes.^3^

**FIGURE 3:**
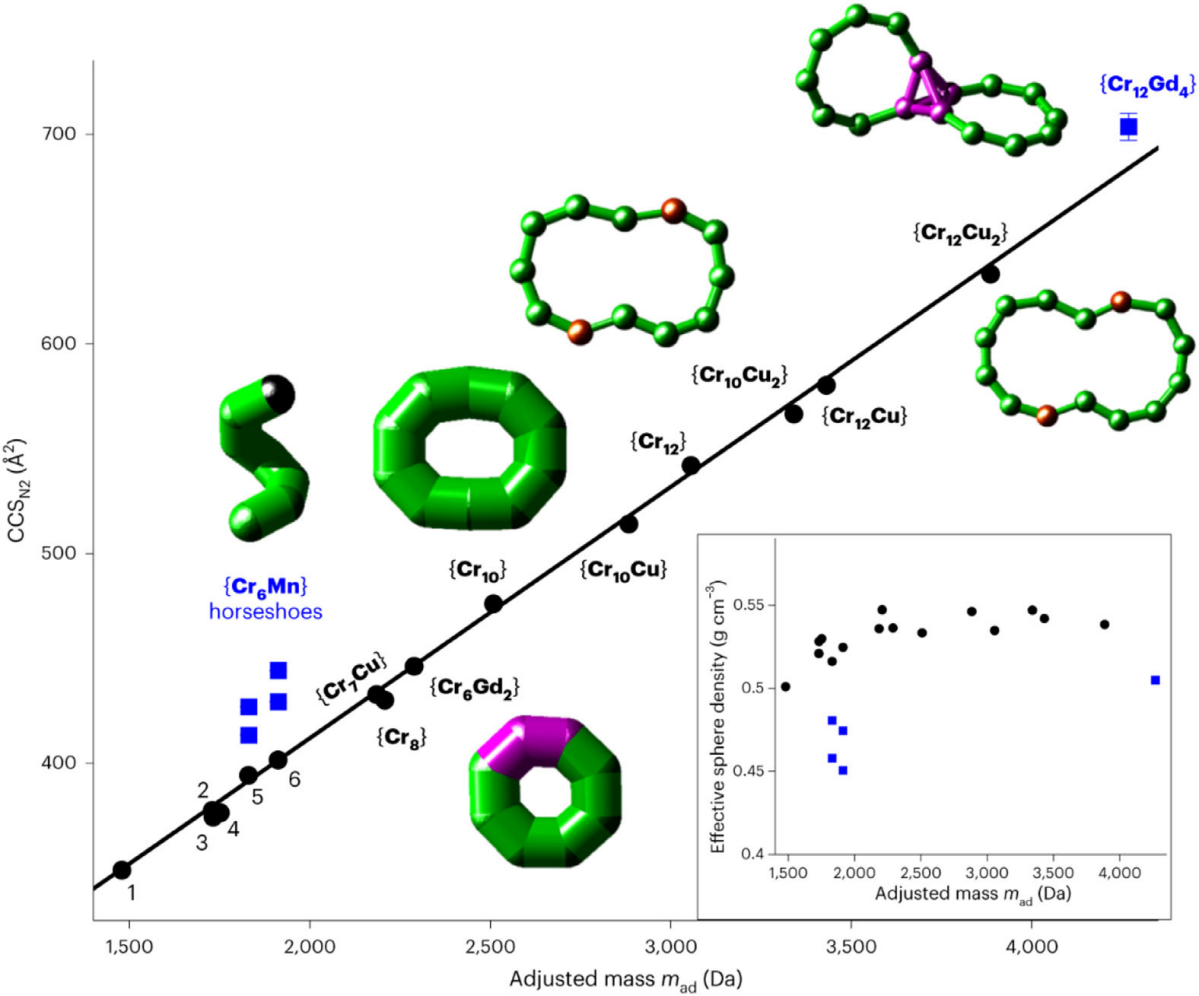
Correlation between collision cross section in nitrogen (CCS_N2_) and adjusted mass (m_ad_) for polymetallic species. The cyclic complexes show a linear correlation (black), which does not hold for acyclic ions (blue, {Cr_12_Gd_4_} and {Cr_6_Mn} horseshoes). 1: {Cr_5_Cu}; 2: {Cr_5_Gd_2_}; 3: {Cr_5_Cu_2_}; 4: {Cr_6_Cu}; 5, 6: {Cr_6_Mn} rings. Inset: Schematics of selected cyclic and acyclic complexes (Cr: green, Cu: brown, Gd: purple, Mn: black). Effective sphere density (packing density) derived from CCS_N2_ values versus m_ad_ (black circles: cyclic, blue squares: acyclic), showing that the acyclic {Cr_6_Mn} horseshoes and {Cr_12_Gd_4_} have a lower effective sphere density than the cyclic species. Reprinted with permission.^4^

I further developed a strategy to form polymetallic rings in the gas phase that are relevant for quantum applications and electronics but are so far impossible to synthesize in solution. The gas‐phase synthesis was achieved via collision‐induced dissociation of larger precursors. (Here, ions are accelerated into a collision cell filled with a neutral gas leading to collisions, which results in the fragmentation to smaller polymetallic species.) On the millisecond timescale of the experiment, these fragments rearrange to smaller closed, cyclic species — as evidenced by IM and the packing density of the complexes (Figure 3). The collision cross section (CCS, informing on size and shape and derived from IM‐MS data) linearly correlates with the ion mass if the latter is cyclic, a relationship that does not hold for acyclic assemblies.^4^ This makes it possible to rapidly characterize the topology of such molecules using only small amounts of sample.

In general, the formation and characterisation of these complexes in the gas phase are relevant for synthetic chemistry since they allow the prediction of which of these species are worth targeting in the bulk phase, based on their abundance and structure in the gas phase.

## 3 WHAT ADVICE WOULD YOU GIVE TO STARTING PhD STUDENTS?

My general advice would be to take a project or job that you really enjoy — life is too short to do things that you do not like, and more often than not there is a choice. Ask as many questions as possible and do not think you have to solve every problem completely by yourself, odds are someone faced the same issue before and can help. PhD work can become lonely, so balancing individual work with collaborations is really helpful and also a lot of fun.

## 4 WHAT DO YOU CONSIDER TO BE THE MORE EXCITING TOPICS IN YOUR FIELD?

I am very excited about the recent developments in the field of MS. For applications, I think the “omics” fields as well as imaging techniques have the potential to be a major game changer in public health. Although I am not involved in this type of research, I am happy to work in the group leading the development of a diagnostic test for Parkinson's disease using MS.^5^, ^6^

I have recently more interested in instrument development, and the progress made by the manufacturers over the last years in the field has been astonishing. From an instrument perspective, I think charge detection mass spectrometry for the analysis of ultra‐large assemblies is a technique of the future. Also, I believe that the fusion of MS with gas‐phase spectroscopy methods will be transformative, provided they become more readily available.

One topic I am particularly interested in, and which I will work on during my postdoctoral research here in Manchester, is the so‐called “ion soft‐landing”. In this technique, gas phase ions are gently deposited on a surface and subsequently analysed with other techniques, for example electron microscopy. By employing microscopy alongside MS, we obtain a substantial amount of structural information at a notably higher resolution, connecting structural data from the gas phase with those in the solution. I am looking forward to designing, building, and applying instrumentation where ion soft‐landing is coupled not just to MS but also to IM.

## 5 WHAT OUTREACH ACTIVITIES DO YOU CONDUCT TO PROMOTE SCIENCE?

Since I left high school, I have been involved in organizing the same chemistry competitions that I benefitted from as a student. For example as an undergraduate student, I have founded a three‐day seminar for the best students of my home state Saxony‐Anhalt, and since it was established, our state has been incredibly successful in securing spots in the German national team. I have been on the Advisory Board of the Friends of the Chemistry Olympiad for 8 years, and since last year I am on the board of the eLeMeNTe society, which is driven to promote science in my home state Saxony‐Anhalt. Here I help organize the society's goals and focus, and also some events from a distance. I am very enthusiastic about outreach and led a regular column in the German chemistry magazine “Chemie in Unserer Zeit” (“*Contemporary Chemistry*”) for 2 years, in which we discussed problems from chemistry competitions for a broader audience.^7^, ^8^, ^9^ I also authored an outreach article on IM‐MS for “Nachrichten aus der Chemie”,^10^ the journal for German Chemical Society members, and have been a reviewer for exam problems for the International Chemistry Olympiad for the past 5 years. I was also a trainer and mentor in my local chess club, as well as a board member of the Youth Chess Society in Saxony‐Anhalt. For the latter, I founded and led a public outreach team of three fellow volunteers.

## 6 WHO WERE THE MOST INFLUENTIAL PEOPLE IN YOUR PATH AS A SCIENTIST?

I have been incredibly lucky to already have been supported and mentored by many fantastic people. My family is full of scientists, so I always had role models and support from within my family — my mum is a mathematician and my dad is a physicist, for example. My chemistry teacher Birgitt Felsche also had a big impact, as she always encouraged me to pursue chemistry in and outside high school. I am also grateful to Frank Edelmann and Volker Lorenz, who hosted me in year 12 of my high school for an internship at Otto‐von‐Guericke University Magdeburg, leading to my first publication.^11^ Afterwards, I had a lot of great fellow students at Leipzig University, and incredible colleagues and mentors who supervised my research abroad, namely Margarita Aliaga (Pontificia Universidad Católica de Chile), Nicole Rijs (University of New South Wales) and Justin Caram (University of California, Los Angeles). These internships have led not only to a couple of papers,^12^, ^13^, ^14^ but also to still‐ongoing collaborations with these groups.^15^ For the last 3 years, I have been mentored by many terrific scientists at The University of Manchester, in particular by my wonderful PhD supervisors Perdita Barran and Richard Winpenny.

## 7 CAN YOU SAY SOMETHING ABOUT YOUR HOBBIES OUTSIDE THE LABORATORY?

**FIGURE 4:**
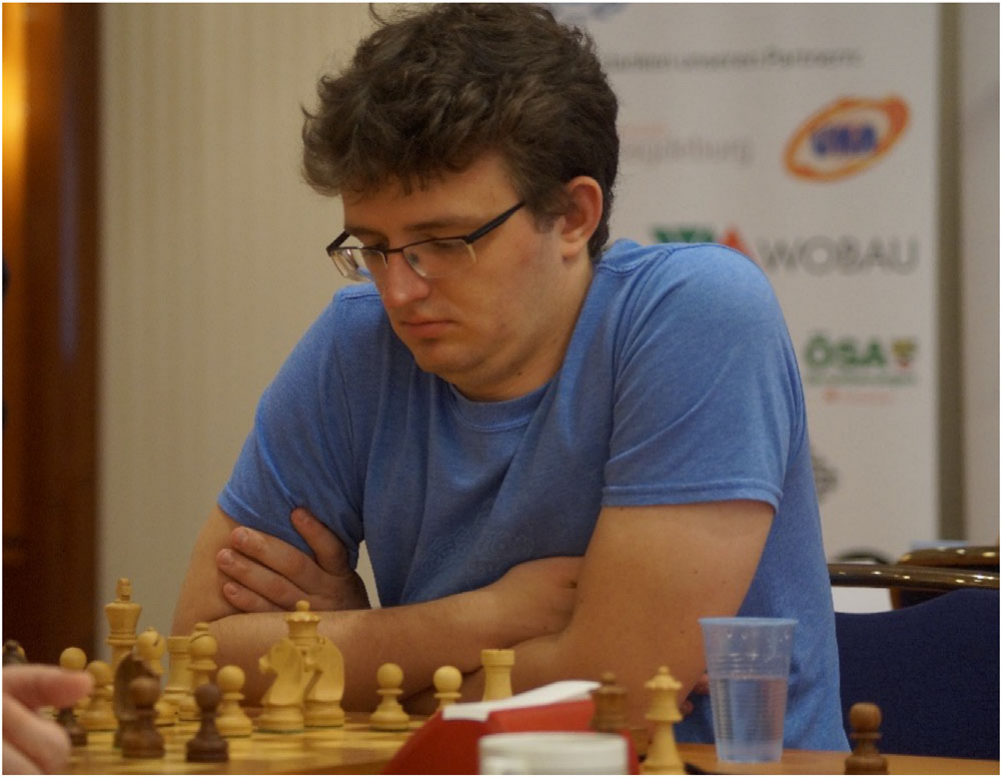
Photo made during the final round of the German Chess Championship 2021 in Magdeburg, Germany. Photo: Frank Hoppe.

For almost two decades, I have played competitive chess on the state and national levels. I was a regular participant in the German Chess Championship in both the youth and open sections (Figure 4), and I would say my biggest success was getting fifth in the under‐18 German chess championship in 2016. Other than that, I enjoy cycling, swimming, meeting friends, and travelling.

## CONFLICT OF INTEREST STATEMENT

The authors declare no conflict of interest.
